# Impregnation of *Melaleuca* Family Essential Oil Nanoemulsions into Pectin:Polyvinyl Alcohol Patches to Provide an Antibacterial Environment for Infected Wounds

**DOI:** 10.1002/open.202500117

**Published:** 2025-06-25

**Authors:** Didem Demir, Seda Ceylan, Semih Latif İpek, Deren Aslan, Veli Özbolat

**Affiliations:** ^1^ Department of Chemistry and Chemical Process Technologies Tarsus University 33100 Mersin Türkiye; ^2^ Department of Bioengineering Adana Alparslan Türkeş Science and Technology University 01250 Adana Türkiye; ^3^ Department of Food Engineering Adana Alparslan Türkeş Science and Technology University 01250 Adana Türkiye; ^4^ Department of Chemistry Dumlupınar University 43100 Kütahya Türkiye; ^5^ Department of Biotechnology Cukurova University 01250 Adana Türkiye; ^6^ Biotechnology Research and Application Center Cukurova University 01250 Adana Türkiye; ^7^ Department of Mechanical Engineering Ceyhan Engineering Faculty Cukurova University 01950 Adana Türkiye; ^8^ Department of Tissue Engineering Cukurova University 01250 Adana Türkiye

**Keywords:** nanoemulsion, niaouli essential oil, pectin, polyvinyl alcohol, tea tree oil, wound dressing

## Abstract

Essential oils have long been utilized in food, cosmetic, and medicinal applications. Recently, their biomedical use for wound healing, skin repair, and tissue regeneration has gained considerable attention. In this study, tea tree oil (TTO) and niaouli oil (NIO) were formulated into aqueous nanoemulsions (NEs) and incorporated into pectin/polyvinyl alcohol (PP) thin films to develop antibacterial wound dressing patches. The NEs were characterized by dynamic light scattering (DLS), and their morphological and chemical structures were also analyzed. The patches’ morphology, hydrophilicity, swelling ratio, and mechanical properties were evaluated to assess the effect of NEs on material performance. Antibacterial activity assessed by plate count and agar diffusion methods against six bacteria commonly associated with infected wounds showed significant efficacy of NEs‐loaded patches against Gram‐negative strains and *Escherichia coli*. Direct and indirect cytotoxicity tests, using Mouse embryonic fibroblast (MEF) cells, confirmed that NEs incorporation maintained cell viability within acceptable limits and promoted their biocompatibility. These findings suggest that TTO and NIO‐based nanoemulsion patches are promising candidates for antibacterial wound dressings.

## Graphical abstract

This study reports the fabrication, characterization, and biological evaluation of bioactive patches made from pectin:polyvinyl alcohol with tea tree and niaouli oil nanoemulsions via solvent evaporation. The patches are flexible, transparent, adhesive, and absorbent. They are biocompatible and show long‐term antimicrobial activity against E. coli and Gram‐positive bacteria, making them suitable for wound infection treatments.

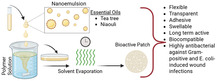

## 1 Introduction

A wound is defined as physical damage that disrupts the normal integrity of the body caused by any agent. This process reduces patients’ quality of life, restricts them socially, increases costs, and affects not only the patient but also society as a whole. Wound healing is a complex process that involves a set of cellular, physiological, and biochemical events, not limited to the wound area, and involving all systems. The incidence of acute or chronic wounds that disrupt skin integrity is increasing day by day, due to the rise in the aging population and chronic diseases such as diabetes, peripheral vascular diseases, and obesity. This situation has led to the proliferation and diversification of new wound treatment methods and options. Especially in recent years, bioactive wound dressings, which have emerged in parallel with the developing material technology, use of bioactive compounds, and nanotechnological approaches, have positively changed the course of treatment.^[^ ^1^, ^2^ ^]^

Dressing materials, which are among the primary treatments for wounds, aim to reduce the risk of infection and secondary injury by rapidly covering the wound. It also helps to provide a moist environment and maintain temperature, provides pain relief and improves hypoxic conditions to promote wound healing. In addition, some types of dressings have the property of not adhering to the tissue and causing less pain to the patient during dressing changes, overcoming the main limitation of traditional dressings. Thanks to their advantages, many studies are being conducted on modern dressings such as foams,^[^ ^3^ ^]^ hydrogels,^[^ ^4^ ^]^ films^[^ ^5^ ^]^ and three‐dimensional printed patterns^[^ ^6^ ^]^ based on biocompatible and biodegradable polymers to solve clinical problems in the treatment of wounds.^[^ ^7^ ^]^ Upon examining the current studies, it is seen that studies on composite patches are intensified by combining polymeric wound dressings with functionalized materials to prevent infections, assist the wound healing process and improve skin restoration.^[^ ^8^ ^]^ This approach aims to avoid wound infection by functionalizing wound dressings with antimicrobial agents such as nanoparticles and antibiotics. In addition, a new field of research has emerged with the use of medicinal plant components, which are very popular in recent times and widely used in traditional wound treatment in different parts of the world, with the advantage of having natural medicinal properties, non‐toxic or less side effects, environmentally sustainable, easily available and less cosmetic.^[^ ^8^, ^9^, ^10^ ^]^

The most common medicinal plant compounds related to the functionalization of wound dressings are essential oils. Essential oils exhibit unique physicochemical properties and diverse biological activities due to the presence of many components such as limonene, α‐pinene, geraniol, menthol, eucalyptol and thymol.^[^ ^11^ ^]^ Studies on essential oils from various plant sources such as tea tree (*Melaleuca alternifolia),* ^[^ ^12^, ^13^ ^]^ thyme (*Thymus vulgaris*),^[^ ^14^ ^]^ lavender (*Lavandula* sp.),^[^ ^15^ ^]^ peppermint (*Mentha piperita*),^[^ ^16^ ^]^ lemon *(Citrus limon)* ^[^ ^17^ ^]^ and eucalyptus *(Myrtaceae)* ^[^ ^18^ ^]^ are well represented in the literature and are recognized to possess a range of biological activities such as antibacterial, antifungal, algicidal, antibiofilm, antioxidant, anticancer, anti‐inflammatory and insecticidal. However, the direct use of essential oils has been severely limited by their non‐optimal properties, such as limited solubility in aqueous media, high volatility and degradability, poor stability, high hydrophobicity and irritation at high concentrations.^[^ ^19^, ^20^ ^]^ Aqueous nanoemulsions (NEs) are among the formulation techniques developed for the efficient dispersion of essential oils due to their advantageous properties such as easy preparation and low production costs. In addition, NEs are considered as potentially effective candidates for applications such as wound healing and surface decontamination. The antimicrobial activity of NEs, unlike classical antibiotics, does not depend on specific targets; this allows broad‐spectrum microbial inactivation while limiting the potential for pathogens to develop resistance.^[^ ^21^ ^]^ Furthermore, the small droplet sizes of NEs facilitate the rapid and effective diffusion of essential oils into the bacterial cell wall, leading to disruption of cell membrane integrity and cell lysis. In addition, essential oils encapsulated in NEs contribute to the denaturation of proteins in bacterial cells, contributing to the loss of both cellular structure and metabolic functions. These mechanisms enhance the interactions between the NEs and microbial cells, thus enhancing antimicrobial activity.^[^ ^22^ ^]^

Especially in the last few years, the effectiveness of essential oils encapsulated as nano‐ and micro‐emulsions by combining different carriers has been investigated in ongoing studies with increasing interest.^[^ ^23^, ^24^ ^]^ For example, in a recent study, Bahloul et al. prepared Tunisian Pituranthos tortuosus essential oil‐based NEs and combined it with the gelling agent Sepimax Zen to investigate the in vivo wound healing effect.^[^ ^25^ ^]^ In another study, Sweet fennel oil NEs were incorporated with chitosan:polyvinyl alcohol films fabricated using solvent casting method to enhance the therapeutic efficacy of the oil and promote tissue regeneration rather than simply wound healing.^[^ ^26^ ^]^ Cai et al. also combined eucalyptus essential oil NEs with a hydrogel matrix based on carboxymethyl chitosan for infected wounds.^[^ ^27^ ^]^

Recently, on the trend of adding essential oils in emulsion form in wound healing applications, we wanted to design wound dressing patches that may have the potential for use, especially in infected wounds by encapsulating the NEs of tea tree oil (TTO), an essential oil frequently studied in the literature, and the more rarely known niaouli (NIO) essential oils in a carrier polymer matrix. As one of the polymer matrix compouns, polyvinyl alcohol (PVA) was selected due to its well‐known biocompatibility, excellent film‐forming ability, and mechanical strength, which are desirable for wound dressing applications.^[^ ^28^ ^]^ As the other compound, pectin, a natural polysaccharide, was incorporated to enhance the biocompatibility and to provide additional bioadhesive properties that can facilitate interaction with the wound bed.^[^ ^29^ ^]^ Tween 80 was used as a surfactant to stabilize the essential oil NEs owing to its proven efficacy in stabilizing hydrophobic compounds in aqueous media and its well‐established safety profile in pharmaceutical applications.^[^ ^30^ ^]^ The encapsulation process of both essential oils in nanoemulsion to enhance their stability and antibacterial activity was morphologically and chemically characterized. The patches formed with NEs formulations added into thin films and these prepared patches were physicochemically characterized and their biological properties were evaluated in terms of antibacterial activities and in vitro cytotoxicty studies.

## 2 Experimental Section

### 2.1 Materials

As essential oils, NIO and TTO used for the preparation of NEs formulations were purchased from a local company (Monoville) in Aydın, Türkiye. Highly esterified citrus peel pectin was supplied from Danisco (Czech Republic) with an esterification degree of 67–71%. Polyvinyl alcohol with 99 + % hydrolyzed (Mw 89,000–98,000), glycerol and Tween 80 were obtained from Sigma‐Aldrich (USA).

### 2.2 Preperation of Nanoemulsion Suspensions (NEs)

The NEs were prepared by the method according to other studies^[^ ^31^, ^32^, ^33^ ^]^ with some modifications. Briefly, 1 mL of NIO and 1 mL of TTO oils were combined with 9 mL of a solvent consisting of surfactant and water (containing 2.5 mL of Tween‐80 and 6.5 mL of distilled water) at a 1:9 volume ratio (oil:solvent). Subsequently, the combination was mixed at 15,000 rpm by a homogenizer (BioBase, BH‐50P, China) for 120 s, rested for 10 s, and then mixed for 120 s to obtain the final NEs suspensions.

### 2.3 Polymeric Dressing Formulation

To prepare the neat films (PP), PEC and PVA were selectedto produce polymeric patcehs. PEC in 5% wt. solution was prepared by dissolving the powder PEC in distilled water at 60 °C until a homogeneous solution was formed. On the other hand, 10% wt. PVA solution was prepared in distilled water at 95 °C under constant stirring. The polymer solutions were mixed at a volumetric ratio of 1:1 in the presence of glycerin (20 μL) as a plasticizer. Finally, ≈3 mL of the resulting clear solution was poured into Teflon dishes. The water solvent was evaporated in an oven at 45 ± 5 °C for 24 h. The resulting polymeric films were peeled off the dishes and used for subsequent analyses.

**Figure 1:**
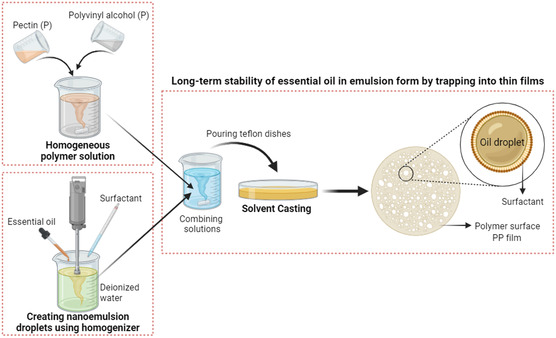
Concept of study by illustrating the experimental steps of NEs incorporated polymer patches.

To prepare the polymeric patches incorporating NEs, 30 μL of each NEs suspension (TTO and NIO) were slowly added dropwise to the polymer mixture, followed by the experimental steps outlined above for neat films. The samples produced in the study were called PP for neat polymer film, NIO@PP for NIO NEs additive, and TTO@PP for TTO NEs additive (**Figure** **1**).

### 2.4 Characterization of NEs

The droplet size distribution of the NEs was determined using the dynamic light scattering (DLS) analyzer method with the Zetasizer Nano ZSP (Malvern Instruments, Worcestershire, UK) at 25 ± 1 °C. The morphological structure of the NEs was analyzed by optical microscopy (Carl Zeiss Axiovert 40 MAT, Germany). The interaction of essential oils as the active ingredient with the excipients in the nanoemulsion formulation was investigated by Fourier transform infrared spectroscopy (FTIR) (Perkin‐Elmer Spectrum 1000, USA). FTIR spectra of NIO, TTO, NIO NEs and TTO NEs suspensions were obtained in the range of 400–4000 cm^−1^ with a minimum of 16 scans at a resolution of 4 cm^−1^.

### 2.5 Characterization of Polymeric Dressings

For morphological evaluation, the digital images of the samples were captured under daylight using a mobile telephone camera. Scanning electron microscopy (SEM) images were obtained using Carl Zeiss microscope (SUPRA 55, Germany). Prior to the imaging process, the samples were coated with a thin layer of platinum using a sputter coating machine. The images were obtained at 5 kV accelerating voltage with 500 and 1,000 kX magnifications.

The swelling ratio of the polymer patches was measured in distilled water at 37 °C in a temperature‐controlled water bath. First, thin films were cut to 1 × 1 cm and the samples were accurately weighed (Wo). Then, the films incubated in distilled water were removed at certain time intervals (5, 15, 30, 60 and 120 min) and the excess water on their surfaces was absorbed on filter paper and weighed again in a swollen state (Ws). The following equation was used for the calculation of the water holding capacity. All experiments were done in triplicate.

(1) Swelling ratio, % wt = [(Ws − Wo)/Wo]*100

In parallel with the swelling ratio, the contact angle was measured to determine the hydrophilicity of the polymer patches. The contact angle measurements were performed on dry film samples at room temperature (≈25 °C) with Theta Lite optical tensiometer (Attension, Biolin Scientific, Sweden). Distilled water was used as the testing liquid, and a droplet volume of 4 μL was applied to the sample surface. The contact angle measurements of the samples were made by taking 15–20 recordings per second for a single distilled water drop with ±1° sensitivity at room temperature.

In order to evaluate the mechanical properties, tensile strength and elongation at break values were determined using a TA‐XT Plus Texture Analyzer (Stable Micro Systems, UK). The films were cut into rectangles measuring 1 cm in width by 5 cm in length. Samples were stretched from a clamping distance of 30 mm at a speed of 10 mm min^−1^, and the tests were repeated for three samples. The thickness of the films was measured by a micrometer from five different points of each film.

### 2.6 Antibacterial Activity

#### 2.6.1 Bacterial Strains and Growth Conditions

Standard cultures for antibacterial assays were procured from the American Type Culture Collection (ATCC), Manassas, VA, USA. The following bacteria were examined in the study of the antibacterial activity of polymer patches which are frequently identified in infected wounds. These were Gram pozitive (+); *Staphyloccocus aureus* (ATCC 29213), methicilin‐resistant *Staphylococcus aureus* (MRSA, ATCC 33592), *Bacillus subtilis* (ATCC 13048) and Gram negative (−); *Escherichia coli* (ATCC 25922), *Pseudomonas aeruginosa* (ATCC 27853), *Acinetobacter baumannii* (ATCC 19606). Nutrient broth and Tryptic Soy Broth (Merck, Germany) were used for the preparation of bacterial culture suspensions and evaluation of the colony forming units (CFU).

#### 2.6.2 Agar Diffusion Method

The agar diffusion method was applied as an alternative means of evaluating the antibacterial efficacy of polymeric patches, as outlined by Liu et al.^[^ ^34^ ^]^ Six bacterial strains were cultured and activated in Tryptic Soy Broth overnight at 37 °C. Prior to inoculation, bacterial suspensions were diluted in sterile saline solution (0.85% w/v NaCl) according to 0.5 McFarland standard (1.5 × 10^8^ CFU mL^−1^). A bacterial suspension of 0.1 mL at 0.5 McFarland standard was spreaded on Mueller‐Hinton agar using sterile cotton swab sticks and the agar was left to dry. Disks with a diameter of 6 mm were cut from UV‐sterilized polymeric patches using a sterile cork borer and positioned on Mueller‐Hinton Agar plates that had been previously inoculated. Ofloxacin (5 μg disc^−1^) was used as postive control (PC). Following a 24 h incubation period at 37 °C, the diameter of the inhibition zone of each disk was measured by repeating three times.

#### 2.6.3 Plate Count Method

Two distinst methodologies were performed to assess the antibacterial properties of the samples. Initally, the polymeric patches were sterilized under UV light in a laminar flow hood. The Plate Count method was employed on the aforementioned bacteria in accordance with a study by Fang et al.^[^ ^35^ ^]^ with minor modifications. Bacterial cultures were harvested upon reaching an OD_6__0__0_ of 0.6. The quantity of 20 mg of each patch was weighed and transferred into sterile centrifuge tubes. 3 mL of bacterial culture medium and 50 μL of six different bacterial suspensions (≈2.4 × 10^7^ cells) were added separately to tubes. Then, the tubes were subjected to an incubation process in a shaker at a temperature of 37 °C for a duration of 24 h. Following the incubation, the solutions were diluted to a concentartion of 10^−5^. A 100 μL portion of diluted bacterial solutions was inoculated on three parallel plates containing plate count agar and spread evenly by using sterile cotton swab sticks. After incubation at 37 °C for 24 h, the colonies were counted to observe an antibacterial effect. Kanamycin (50 μg mL^−1^) was applied as the PC for each bacterial culture. The enumeration of colonies was conducted using the ImageJ 1.53t version (ImageJ, USA). All experiments were conducted triplicate.

### 2.7 Biocompatibility Studies

#### 2.7.1 Cell Culture

MEF (mouse embryonic fibroblasts, CF‐1) (ATCC SCRC‐1040) were cultivated in DMEM, with the addition of 10% foetal bovine serum (FBS) and 55 μM 2‐mercaptoethanol. The cell line was cultivated in an incubator maintained at 37 °C, with a relative humidity of 90–95% and a carbon dioxide concentration of 5%. Once the cells had reached 80% confluence, they were passaged using trypsinization at three‐day intervals.

#### 2.7.2 Direct Cytotoxicity Assay

The polymer patchs were cut into equal‐sized pieces with a measurement of 4 mm in length and 1 mm in width. After that, the patches were exposed to UV for 2 h, sterilized and then placed within a 24‐well culture plate. Before seeding process, DMEM was added to the patches and left to incubate for a period of time to penetrate the medium into patches. 2 × 10^4^ cells in 1 μL of DMEM culture medium were seeded in the center of patch samples and also in wells without polymer patch (control). The samples were kept at incubator for 2 h. Following this period, an equal amount of DMEM (1 mL) was added on samples and they were incubated for 24 and 48 h in a humidifed incubator at 37 °C with 5% CO_2_. Thereafter, MTT (3‐(4,5‐dimethylthiazol‐2‐yl)‐2,5‐diphenyltetrazolium bromide) analysis was conducted which was employed to measure the viability of the cells at 570 nm using a microplate reader (Spectrostar Nano, BMG Labtech, Ortenberg, Germany). The experiments were conducted in triplicate.

#### 2.7.3 DAPI Protocol

This protocol was implemented by taking into account the method used by İpek et al.^[^ ^36^ ^]^ to observe cells attaching to polymeric patches. Following incubation periods of 24 and 48 h, the cell culture medium was removed, and the cells underwent two PBS (phosphate‐buffered saline) washes. Subsequently, 4% paraformaldehyde solution was added to each well and left at room temperature for 20 min. The cells were then washed twice with PBS. PBS containing 0.1% Triton X‐100 was added to the cells to permeabilize the cell patches and left for 10 min. PBS was used to wash the cells twice. PBS containing 1% bovine serum albumin (BSA) was used to dilute DAPI 1:1000. DAPI solution was applied to the wells to label the nuclei and left at room temperature for 30 min in the dark. The cells were then washed twice with PBS and BSA. Finally, a Leica DM IL LED inverted microscope (Leica Biosystems, Wetzlar, Germany) was used to view the cells under fluorescent illumination.

#### 2.7.4 Indirect Cytotoxicity Assay

First, the degradation products to be used in the indirec*t* test were prepared. For this, the polymeric patches were cut into small pieces (1 × 1 cm) and exposed to UV for 2 h. Then, they were placed in a sterile falcon tube and 2 mL of medium (DMEM + 10% FBS) was added. Then, the falcon tubes were placed in a 37 °C water bath for 24 and 48 h to allow the formation of indirect degradation products. While test groups were given media incubated with polymeric patches, the control group was given 2 mL DMEM + 10% FBS directly. For the 24 and 48 h incubations, 12 × 10^3^ MEF cells were seeded into wells and placed in a 37 °C humidified incubator with 5% CO_2_. Following the incubation period, cell viabilities were assessed using the MTT assay. The assays were conducted in triplicate, and cell viabilities were determined relative to the control groups. Additionally, cell observation occurred at 24 and 48 h using an inverted microscope. Subsequently, the MTT cell viability assay was conducted using the 24‐well plates. Absorbances were measured at 570 nm using a microplate reader (Spectrostar Nano, BMG Labtech, Ortenberg, Germany) to evaluate the cytotoxic effects of patches degradation products.

## 3 Results and Discussions

### 3.1 Physicochemical Examination of NEs

**Figure 2:**
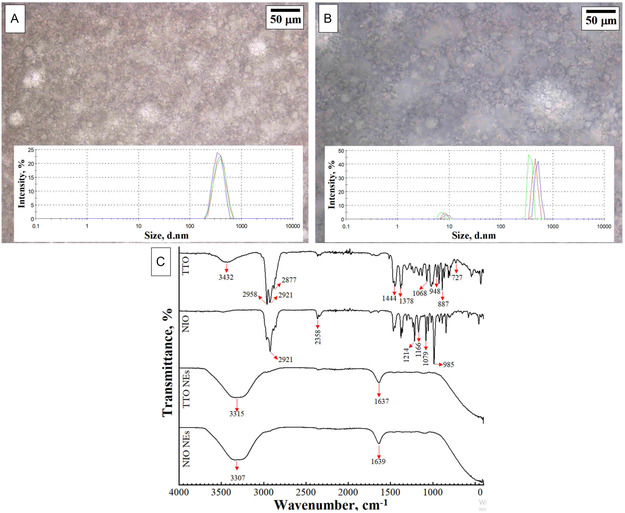
Characterization of nanoemulsions (NEs): A) Optical microscopy and DLS analysis of TTO NEs; B) Optical microscopy and DLS analysis of NIO NEs; C) Chemical profiles of essential oils (TTO, NIO) and their nanoemulsions (TTO NEs, NIO NEs).

To overcome problems such as overdosing, hydrophobicity, high volatility, and poor stability that can occur when essential oils are used directly, NEs formulations were prepared and characterized. It is also assumed that nanoemulsions, due to their nano‐sized droplets that increase the active surface area, have superior antimicrobial activity than conventional emulsions with significantly higher droplet size.^[^ ^37^ ^]^ In the context of the current study, NIO and TTO essential oils, which are known to possess high antibacterial activities, were selected to produce emulsion suspensions. The obtained suspensions were initially evaluated by optical microscopy images and particle size analysis obtained by DLS measurements. **Figure** **2**A,B show the optic microscope images of TTO and NIO NEs and the particle size distribution graphs captured in the images, respectively. According to morphological evaluation, it was found that the roughly spherical‐ shape of the nanoemulsion droplets for TTO and NIO NEs suspensions was noticed as demonstrated in Figure 2A,B. On the other hand, determination of the particle size and distribution of the emulsions is very important for the stability of the microemulsion during storage and its dispersibility in the application.^[^ ^38^ ^]^ When the particle size distribution histograms obtained according to DLS measurements are analyzed, it is seen that TTO NEs particles show a more homogeneous distribution than NIO NEs. For TTO NEs, the average diameter and polydispersity index (PDI) were found as 384.40 ± 16.50 nm and 0.44, respectively. For NIO NEs, the average diameter and PDI were found as 445.60 ± 74.53 nm and 1.0, respectively. formulations, which depend on different factors such as composition, time and temperature. The PDI value ranges from 0 to 1.0, where 0 represents a monodisperse system and 1.0 indicates a polydisperse particle distribution. The PDI value obtained for TTO NEs is less than 0.5, which is defined as a more homogeneous and stable structure of the nanoemulsion as previously indicated in other studies.^[^ ^39^, ^40^ ^]^ The NIO NEs suspension produced under the same experimental conditions has a more heterogeneous structure with a wide particle diameter distribution.

In addition to the morphological characteristics of the NEs suspensions formed from TTO and NIO essential oils, FTIR analysis was used to interpret possible changes in their chemical structure. In Figure 2C, the FTIR spectra of TTO, NIO, NIO NEs and TTO NEs are shown by labeling the characteristic peaks. The FTIR spectra showed the presence of specific functional groups for the compounds of both essential oils and these characteristic components were commonly identified by numerous peaks between the wavenumber of 600 and 1500 cm^−1^. For TTO (*Melaleuca alternifolia*), three sharp peaks of strong intensity at 2958, 2921 and 2877 cm^−1^ in the spectrum correspond to the stretching vibrations caused by the presence of —C—H bonds in almost all components of TTO. Similarly, the bending vibrations of =C—H bonds caused the formation of sharp peaks at 727, 887 and 948 cm^−1^. In addition, —C—H bending vibrations in compounds such as α‐Pinene, 4‐Carene, Terpinen‐4‐ol, Alloaromadendrene, α‐Bulnesene and naphthalene derivatives such as Cadinene and Calamenene contributed to the formation of sharp peaks seen at 1378 and 1444 cm^−1^. The broad peak at 3432 cm^−1^ was a result of the contribution of the ‐OH groups of Eucalyptol, Terpinen‐4‐ol, and α‐Terpineol. Furthermore, the alcoholic C—O bonds in these compounds also contributed to the formation of a sharp peak at 1068 cm^−1^.^[^ ^41^, ^42^ ^]^ In the spectrum of NIO (Melaleuca viridiflora), more distinct peaks were additionally observed at 1214, 1166, 1079 and 985 cm^−1^, which can be attributed to the C—H stretching vibrations of aromatics.

The FTIR analysis of NEs demonstrated the absence of distinct and sharp peaks in the essential oils. However, a distinct and sharp peak was observed in the NIO and TTO NEs, with the peak occurring at 3307 and 3315 cm^−1^, respectively, indicative of a hydrophilic interaction. This observation aligns with the findings of Kumari et al. who also reported a similar FTIR spectrum shift in thymol essential oil and its nanoemulsion.^[^ ^43^ ^]^

### 3.2 Characterization of Composite Patches

**Figure 3:**
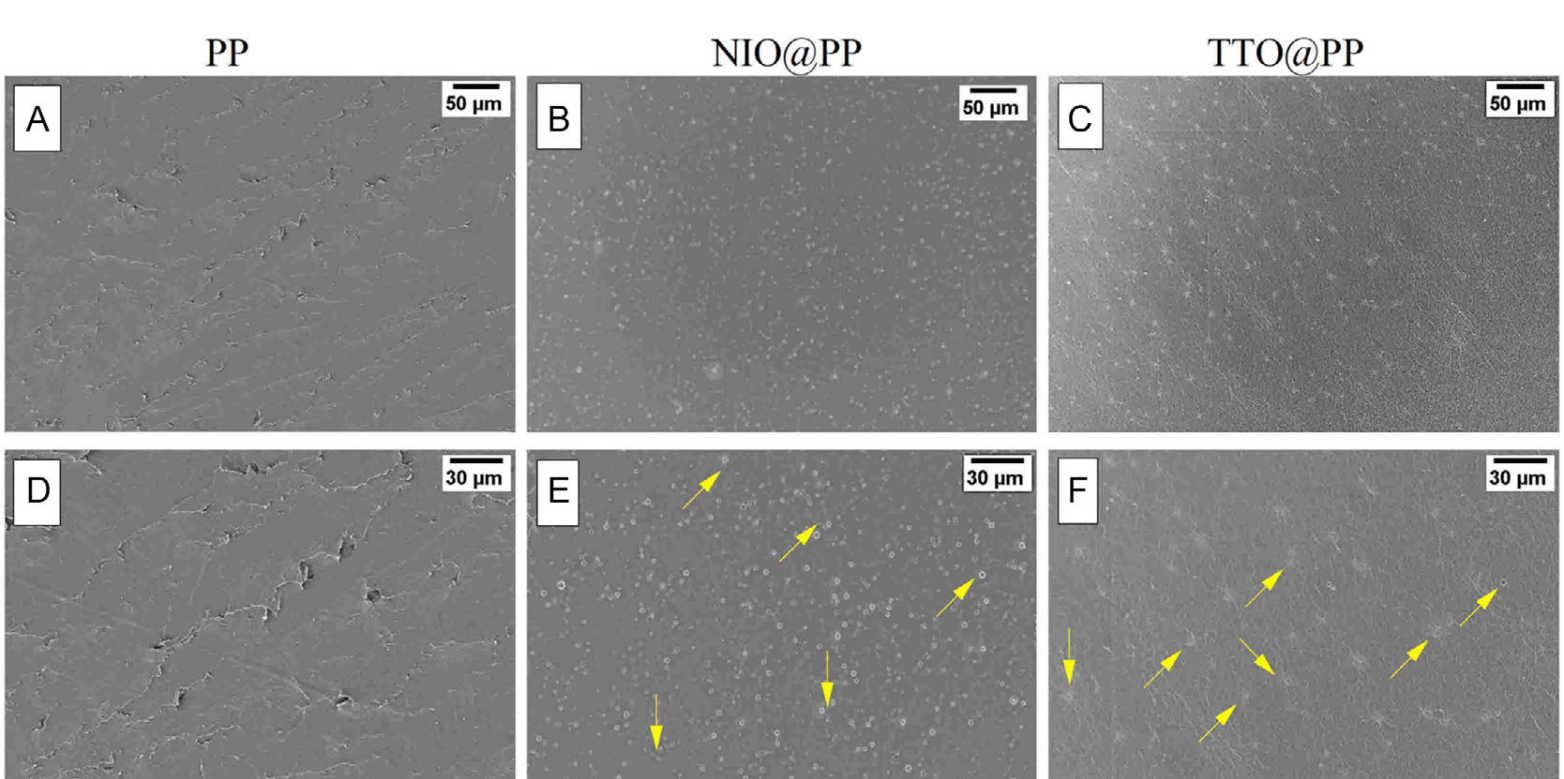
SEM images of the polymer patches: A,D) neat polymer patch (PP); B,E) polymer patch loaded with NIO NEs (NIO@PP); and C,F) polymer patch loaded with TTO NEs (TTO@PP). Scale bars: 50 μm (A–C); 30 μm (D–F).

TTO and NIO NEs suspensions, whose preliminary characterization was completed in the previous part, were entrapped in PP films at equal concentrations. Essential oil NEs, which are widely used in the food industry,^[^ ^44^, ^45^, ^46^ ^]^ are still a very new research area in biomedical applications, especially in the design of composite materials produced by the integration of essential oil NEs into polymeric biomaterials. For this reason, the NEs‐loaded wound dressing patches that we aim to produce in our study were first physicochemically characterized. **Figure** **3** shows the SEM images of PP, NIO@PP and TTO@PP composite patches obtained at different magnifications. The neat polymer film (PP) exhibited a relatively rough surface characterized by microcracks and fractures due to internal stresses and shrinkage during the solvent casting method (Figure 3A,D). After the addition of NIO and TTO NEs to the polymer solution, a significant improvement in the surface morphology of the composite films was observed. Both NIO@PP and TTO@PP films exhibited smoother and more homogeneous surfaces compared to the neat polymer film; cracks and surface defects were found to have disappeared (Figure 3B–F). It is thought that this improvement may be related to the glycerol‐like plasticizing effect of the essential oil emulsions,^[^ ^47^ ^]^ which can be increased the flexibility of the polymer network structure and reduced the internal stresses during film formation. In addition, it was observed that the emulsion droplets were homogeneously distributed in the polymer matrix in both NIO@PP and TTO@PP patches (Figure 3B–F). In both formulations, the NEs droplets were clearly detected and showed a stable distribution within the polymer structure. However, while the droplets were observed to be more distinct and separate on the surface in NIO@PP films (Figure 3B,E), it was observed that the droplets were more integrated into the polymer matrix in TTO@PP films (Figure 3C,F).

**Figure 4:**
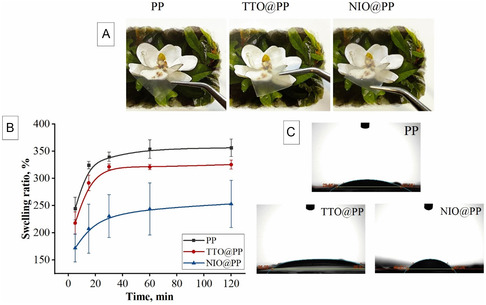
A) Photographs of polymer patches demonstrating their transparency; B) swelling ratio of the polymer patches, and C) contact angle measurements of the polymer patches.

In **Figure** **4**A, the photographs of the thin films under daylight were presented. The photographs taken under a colored background show that the composite films (TTO@PP and NIO@PP) are transparent like neat PP films. After the addition of both the NIO NEs and the TTO NEs to the PP structure, the polymer patches retained their physical appearance, which is particularly important for the examination of the wound without removing the dressing and for better monitoring the healing process.^[^ ^48^, ^49^ ^]^ It was noticed that neat PP and NEs incorporated PP composite patches exhibited hydrogel properties in contact with water and swelled on a volume basis in a very short time, and showed a high swelling ratio. This behavior is also an important characteristic for absorbing exudate and maintaining a moist environment during the wound‐healing process.^[^ ^50^ ^]^ In this context, the swelling ratio of polymeric patches incubated in water at certain time intervals was determined by weight. In Figure 4B, the time‐dependent change in weight is presented up to the equilibrium swelling point. The swelling ratio of PP, TTO@PP, and NIO@PP in the first 5 min was 244.58 ± 20.83%, 217.96 ± 20.68%, and 172.03 ± 25.70%, respectively. When the polymeric patches were compared within themselves, the PP film exhibited the highest swelling ability, and all the films reached the equilibrium water retention point in about 60 min. Although it varies depending on the polymer composition and amount, Kraskouski et al. obtained values ranging between 31 and 72% for the 1 h water retention capacity of PVA and PEC blended films.^[^ ^51^ ^]^ In our study, the high swelling ratio of films can be explained by the presence of PEC, one of the main polymers forming the patch. The PEC may have caused an increase in hydrophilicity, and thus, a higher water retention may have been observed in the films. Similarly, Oh et al. reported an increase in swelling ratio with the addition of PEC to the recipe compared to plain PVA hydrogels.^[^ ^50^ ^]^ After the addition of the NEs, there has been some reduction in the swelling ratio of the films. The addition of NIO NEs and TTO NEs is predicted to reduce the water binding ability of the hydrogel films. Ghasemlou et al. have suggested that this is due to the hydrophobic nature of essential oils, which directly affects the water retention properties of the films.^[^ ^52^ ^]^ Comparing the NIO@PP and TTO@PP samples, it is observed that TTO@PP exhibits a behaviour closer to the PP control thin film. Considering the particle distributions (more homogeneous, stable, and smaller particle diameter) shown in Figure 2A and better penetration into the polymer structure as shown in Figure 3F, TTO NEs suspension did not cause a significant change in the polymer network. However, both of the composite dressings with added NEs showed a high and long‐term water holding capacity similar to that of PP control dressings, an advantage that may help to maintain a moist environment in wounds and promote collagen production and autoimmune repair.

The swelling of thin films by taking water may also be related to the surface properties of the material. Therefore, the contact angle values of the thin films against water were determined according to the photographs obtained in Figure 4C. The contact angle values were measured as 26.40 ± 3.25°, 38.08 ± 1.79°, and 46.44 ± 3.84° for samples PP, TTO@PP, and NIO@PP, respectively. The results obtained here can be evaluated as the distribution of NEs droplets in polymer films, affecting the surface properties and therefore the contact angle values. In the SEM images given in Figure 3, the more pronounced presence of emulsion droplets on the surface in NIO@PP films may have increased the hydrophobic character on the surface and caused the water contact angle to increase slightly. On the other hand, the embedding of droplets in the polymer matrix in TTO@PP films may have reduced the effect of the oil phase on the surface and led to a lower contact angle closer to the neat sample. It was observed that these differences were effective on the wettability of the surface, depending on the exposure of essential oils on the surface and the degree of integration into the polymer matrix. In addition, since the surfactant (Tween 80) adsorbed on the interface and reduced the surface or interfacial tension, it may have led to the formation of a strong hydrophilic molecular layer on the surface of the TTO NEs droplets, which were evenly distributed by embedding in the polymer matrix.^[^ ^53^ ^]^

The mechanical properties of polymeric patches to be used as wound dressings are important in terms of the ease of application of the product, its ability to maintain its integrity during use and comfort of use. In addition, mechanical strength can act as an effective barrier by protecting the wound microenvironment against external factors. Therefore, the mechanical characterization of polymer films was evaluated with mechanical properties such as tensile strength and percntage elongation at break. As seen in **Table** **1**, PP film showed the highest strength and elongation at break. It can be said that PP film is more flexible by showing higher tensile strength and elongation compared to TTO@PP and NIO@PP films. These findings can be explained by the decrease in elasticity and increase in plasticity with the inclusion of nanoemulsions, as reported by Almasi et al. for pectin films incorporated with *Origanum majorana* essential oil loaded nanoemulsions.^[^ ^54^ ^]^ Moreover, a similar trend was found in another study conducted by Baghi et al. for pectin film containing nanoemulsified *trans*‐Cinnamaldehyde.^[^ ^55^ ^]^ In addition, an increase in thickness was observed in film thickness measurements made with a micrometer due to increasing oil content. Similar results were also encountered in gelatin‐guar gum films loaded with *Nigella sativa* nanoemulsion produced by Mutlu^[^ ^56^ ^]^ and clove essential oil nanoemulsion‐loaded chitosan films studied by Rui et al.^[^ ^53^ ^]^

**Table 1:** Thickness and tensile properties of polymer patches.

Sample	Thickness [mm]	Strength [Mpa]	Elongation at break [%]
PP	0.16 ± 0.01	4.36 ± 0.49	66.86 ± 8.58
NIO@PP	0.23 ± 0.01	3.82 ± 1.08	34.52 ± 8.20
TTO@PP	0.22 ± 0.02	2.57 ± 0.03	31.45 ± 8.33

### 3.3 Antibacterial Activity

NIO and TTO belong to the *Melaleuca Oil* family and are complex bioactive agents with more than 100 species of sesquiterpenes, monoterpenes, and related alcohols.^[^ ^57^, ^58^ ^]^ These essential oils have strong antibacterial activity against a wide spectrum of pathogenic properties due to their rich natural content.^[^ ^32^, ^59^, ^60^ ^]^ The objective of our study is to demonstrate the efficacy of nanoemulsifying essential oils in comparison to direct embedding into a polymeric system. Nanoemulsifying essential oils has the potential to provide a longer‐lasting and more controlled release, which is a significant advantage over direct embedding. Previous studies have also highlighted that NEs formulations of essential oils may exhibit higher biological properties than direct use of essential oils due to the small particle size, which can result in large contact surfaces and high diffusion levels.^[^ ^32^ ^]^ Based on this idea, we created NEs formulation of two different essential oils from the same family and added them to PP films that have ideal properties as a wound dressing material.

**Figure 5:**
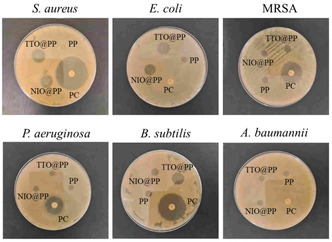
Microbial analysis of the polymeric patches: Representative images of the agar diffusion method. PC: Positive control (Ofloxacin).

In this study, two methods were used for evaluating the antibacterial properties of the patches, agar diffusion and plate counting, against 6 bacteria most of related with infected wounds. Bacterial strains commonly associated with wound infections were selected for antimicrobial testing. *S. aureus*, MRSA, *E. coli*, *P. aeruginosa*, and *A. baumannii* are frequently implicated in acute, chronic, and nosocomial wound infections.^[^ ^61^, ^62^, ^63^ ^]^ *Bacillus subtilis* was included as a model Gram‐positive strain to broaden the antimicrobial assessment.^[^ ^64^ ^]^ As shown in **Table** **2** and **Figure** **5**, the PP patch does not have an antibacterial effect compared to TTO@PP and NIO@PP patches. In addition, it is seen that TTO@PP and NIO@PP patches are not effective against *P. aeruginosa* and *A. baumannii* bacteria strains. The lack of antibacterial activity against these bacteria may be attributed to the outer membrane barrier of Gram‐negative bacteria, which limits the penetration of hydrophobic components of essential oils. Furthermore, the limited diffusion of the active compounds in the agar medium in the disc diffusion assay may have reduced the detectable activity against these strains. For this reason, in the continuation of the study, all samples were evaluated by applying the plate count method against all bacterial strains.

**Table 2:** Inhibition zone diameters (mm ± SD) of the polymeric patches against different microorganisms. (*n* = 3).

Samplea)	*E. coli*	*S. aureus*	MRSA	*P. aeruginosa*	*B. subtilis*	*A. baumannii*
PC	–	36.18 ± 2.51	19.24 ± 1.25	17.60 ± 3.90	22.14 ± 3.64	–
PP	–	–	–	–	–	–
NIO@PP	20.12 ± 5.67	18.30 ± 4.25	7.80 ± 1.21	–	7.60 ± 3.11	–
TTO@PP	22.10 ± 4.55	19.58 ± 3.89	8.25 ± 6.3	–	10.12 ± 2.74	–

a) PC: positive control (Ofloxacin).

**Figure 6:**
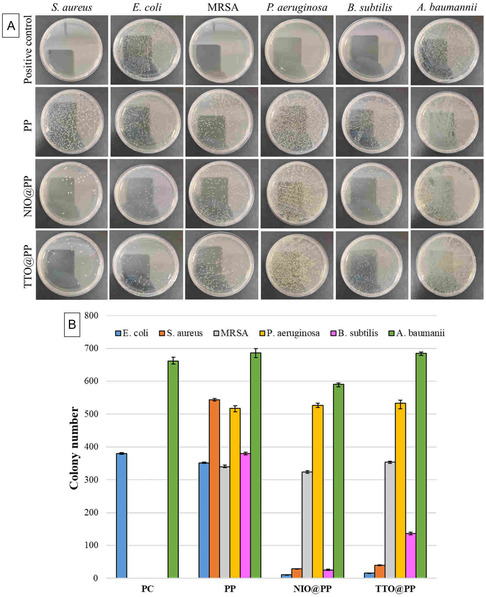
Antibacterial analysis of the polymeric patches: A) Representative images of the plate counting method; B) bar graph showing the number of microbial colonies obtained from the tests. (*n* = 3). PC: Positive control (Kanamycin).

The results presented in **Figure** **6** show that the PP patch did not show any significant antibacterial activity against bacteria. In contrast, TTO@PP and NIO@PP patches provided significant inhibition especially against Gram‐positive bacteria (*S. aureus*, MRSA and *B. subtilis*). This can be interpreted as essential oil nanoemulsions may be more effective especially against Gram‐positive bacterial cell walls. In addition, significant antibacterial effects of both patches were observed against Gram‐negative bacteria such as *E. coli*. However, patches containing TTO and NIO were found to be ineffective against *P. aeruginosa* and *A. baumannii* strains. This can be explained by the fact that the outer membrane structures and intrinsic resistance mechanisms of these bacteria prevent the essential oil components from passing into the cell. The results show that TTO and NIO loaded patches can be evaluated as a potential antimicrobial agent especially against Gram‐positive and E. coli‐induced wound infections.

### 3.4 Cytotoxicity Evaluation

**Figure 7:**
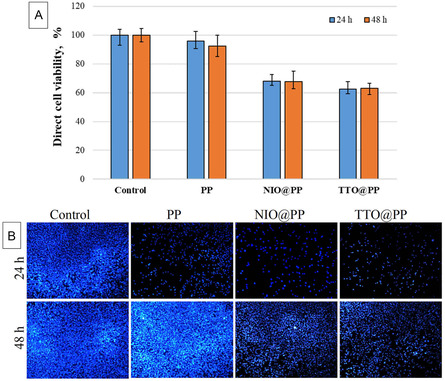
Direct cytotoxicity assessment of polymer patches: A) Cell viability of cells treated with PP, NIO@PP, and TTO@PP polymer patches after 24 and 48 h of incubation. B) DAPI staining images of cells treated with PP, NIO@PP, and TTO@PP polymer patches after 24 and 48 h of incubation.

MEF cells were used for cytotoxicity on PP, TTO@PP and NIO@PP polymer patches. **Figure** **7**A, direct cytotoxicity test shows the NEs added patches’ cell viability, which was found to be higher than %60. In Figure 7B, the cells’ DAPI picture is also displayed. DAPI staining was employed to assess the nuclear morphology of MEF cells at 24 and 48 h. Typically, when these cells are well spread on a surface, their nuclei exhibit an elliptical shape. As depicted in Figure 6, a higher number of cells adhered to the neat PP patches, and these cells proliferated in closer proximity to one another compared to those on other substrates. On control patches, the increased cell density led to nuclei that appeared more elongated than those observed in other groups. Notably, no signs of nuclear condensation or fragmentation were observed on any of the patches. Although cell density was lower on NIO@PP and TTO@PP patches, the nuclei maintained their typical shape. The most significant nuclear morphological changes were evident in cells cultured on TTO@PP patches at both 24 and 48 h. During the initial 24 h, cells on TTO@PP patches failed to adhere, spread, or proliferate effectively, resulting in nuclei that were predominantly circular in shape.

**Figure 8:**
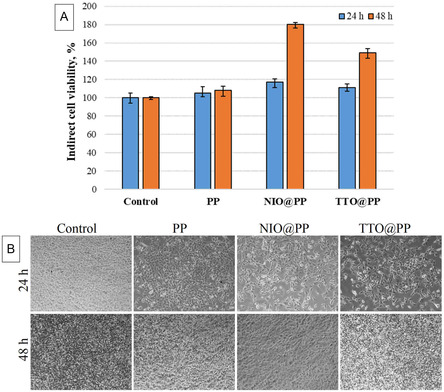
Indirect cytotoxicity assessment of polymer patches: A) Cell viability of cells treated with PP, NIO@PP, and TTO@PP polymer patches after 24 and 48 h of incubation. B) Optical images of cells treated with PP, NIO@PP, and TTO@PP polymer patches after 24 and 48 h of incubation.

**Figure** **8**A,B show the results of the indirect MTT assay and the microscopic images of cells exposed to the fabricated PP@NIO and PP@TTO patches at different time points, respectively, illustrating cell viability and morphology. As it can be seen, there is significant difference between the cell viability of control group and NIO@PP and TTO@PP patches samples at different days (Figure 8A). The NIO@PP patches showed better cell proliferation rates than the control group and TTO@PP film. It was reported that the patches with NIO and TTO enhanced adhesion and proliferation of MEFcells for indirect conditions. An inclusive spectrum of positive biological activity of these oils with respect to the human body largely results can be illustrated the anti‐oxidative effects of various compounds. In addition to this, oils includes also active compounds such as; consists of 1,8‐cineole (oxide) (monoterpene), limonene (monoterpene), a‐pinene (monoterpene), ß‐pinene (monoterpene), and viridiflorol (sesquiterpene) in varying compositions.^[^ ^65^ ^]^ According to reports, 1,8‐cineole (around 55%) is the functional component/biomarker chemical of NIO. This compound may be in responsible for NIO's therapeutic activities, including its capacity to fight bacteria in vitro.^[^ ^32^ ^]^

However, the indirect cell proliferation rates for TTO@PP and NIO@PP polymer patches (medium with TTO@PP and NIO@PP polymer patch degradation products) were higher compared to the direc*t* test (standard medium). Such a behaviour has not been reported elsewhere and needs further investigations. This might be due to the lack of adherence of cells to TTO@PP and NIO@PP polymer patchs because of the oil drops on film surface. Various investigations presents that TTO and NIO incorporated polymeric scaffold incubated one day in medium and results show that cell viability increased with this medium.^[^ ^65^, ^66^ ^]^ Recent studies also show that loading essential oil in polymeric scaffolds affects cell viability in positive direction.^[^ ^67^ ^]^

## 4 Conclusion

In this study, NEs formulations prepared with TTO and NIO essential oils, both belonging to the same biological class, were successfully incorporated into PVA and PEC‐based thin film polymer patches for potential wound dressing applications. TTO NEs were embedded within the polymer matrix, whereas NIO NEs primarily remained on the polymer surface. Following NEs incorporation, the thin films retained key properties relevant to wound care—including transparency, flexibility, adhesion, and swelling ratio—at acceptable levels. Importantly, the composite polymer patches exhibited strong antibacterial activity against both Gram‐positive bacteria and E. coli, attributed to the inherent antimicrobial properties of the essential oils. Although cytotoxicity studies indicated that cell viability in the NEs‐loaded films was slightly lower than in the control, degradation product analysis confirmed that this was not due to the NEs formulation itself. Rather, the 2D film structure may have limited cell adhesion. Therefore, future work may focus on integrating NEs formulations into 3D scaffolds, such as cryogels, to enhance cell attachment, proliferation, and overall biocompatibility. Additionally, the influence of particle size within NE formulations on biological responses warrants further investigation. Given their antibacterial activity against bacterial strains related with infected wounds and favorable preliminary characteristics, these composite polymer patches demonstrate strong potential for use in infected wound treatment and form a promising basis for further optimization and development.

## Conflict of Interest

The authors declare no conflict of interest.

## Author Contributions

**Didem Demir**: design of the study, investigation, methodology, chemical and analytical research, data curation and writing—original draft; **Seda Ceylan**: analysis and interpretation of the data, writing—original draft; **Semih Latif İpek**: methodology, analysis; **Deren Aslan**: methodology and analysis; **Veli Özbolat**: writing—review and editing. All authors have read and agreed to the final version of the manuscript.

## Data Availability

The data that support the findings of this study are available from the corresponding author upon reasonable request.
